# Avoiding the Medial Brachial Cutaneous Nerve in Brachioplasty: An Anatomical Study

**Published:** 2010-01-29

**Authors:** Saeed Chowdhry, Joshua B. Elston, Todd Lefkowitz, Bradon J. Wilhelmi

**Affiliations:** University of Louisville, Louisville, Kentucky

## Abstract

**Objective:** With more patients undergoing bariatric surgery procedures, there has been an increased demand on plastic surgeons to manage excess skin around the body from massive weight loss. The upper arm is one of the areas that require surgical attention. One of the complications of brachioplasty is injury to cutaneous nerves of the arm. We report our findings of the location of the medial brachial cutaneous nerve on the basis of anatomical landmarks to aid the reconstructive surgeon in planning his or her operative approach and procedure. **Methods:** Eight fresh cadaver arms were dissected under loupe magnification. The brachial plexus was dissected from proximal to distal to evaluate the branching points of the cutaneous nerves. Measurements were taken from the medial epicondyle to cutaneous branches off the main nerve. **Results:** At about 7 cm proximal to the medial epicondyle, there is an arborization of 2 to 3 cutaneous branches. This nerve sends 3 to 4 branches through the muscular fascia across the ulnar nerve to skin of the medial arm at about 15 cm proximal to the medial epicondyle. In most cadavers, this was found in the midportion of the arm. **Conclusions:** The plastic surgeon will be challenged to effectively manage excess skin from weight loss. Placing the incisions more posteriorly on the arm will help avoid morbidity associated with injury to these nerves, while still providing an acceptable aesthetic outcome. Knowledge of the anatomy of the course of the medial brachial cutaneous nerve can help the surgeon better plan his or her operative approach to maximize aesthetic benefit and limit nerve injury.

Between 2000 and 2004, the number of brachioplasty operations performed in the United States increased significantly.^1^ With more patients undergoing bariatric surgery procedures, there has been an increased demand on plastic surgeons to manage excess skin around the body from massive weight loss.

The upper arm is an area that requires surgical attention. Old and new techniques such as major brachioplasty, modified brachioplasty, and minibrachioplasty with liposuction address this problem area.^2^^-^5 Ultimately, the degree of excess skin and skin laxity plays a role in determining what type of approach should be undertaken.

One of the complications of brachioplasty surgery is injury to cutaneous nerves of the arm. Most patients in a previous study had not volunteered sensory loss information, because none had been asked about it.^6^ Knoetgen and Moran^7^ describe complications at the Mayo Clinic and report close to a 5% injury rate to cutaneous nerves of the arm during brachioplasty surgery. They describe this type of sensory loss as a major morbidity because it persisted for more than 1 year and can indirectly lead to the formation of pressure ulcers or painful neuromas over the anesthetic area.

Most brachioplasties place the incision medially on the arm to minimize the visibility of the scar. While this sufficiently hides the scar for most patients, the incision and subsequent flap elevation risk damage to cutaneous nerves in this area. Both the medial brachial cutaneous (MBCN) and medial antebrachial cutaneous (MACN) nerves arise from the medial cord of the brachial plexus and travel through the arm to their respective areas of distribution. Knoetgen and Moran (2006) 7have previously described the anatomic branching and arborization of the MACN. However, accurate measurements of MBCN have yet to be described. The surgeon aims to achieve favorable aesthetic outcome and minimize complications. We report our findings of the location of the MBCN on the basis of anatomical landmarks to aid the reconstructive surgeon in planning his or her operative approach and procedure.

## METHODS

**Figure 1:**
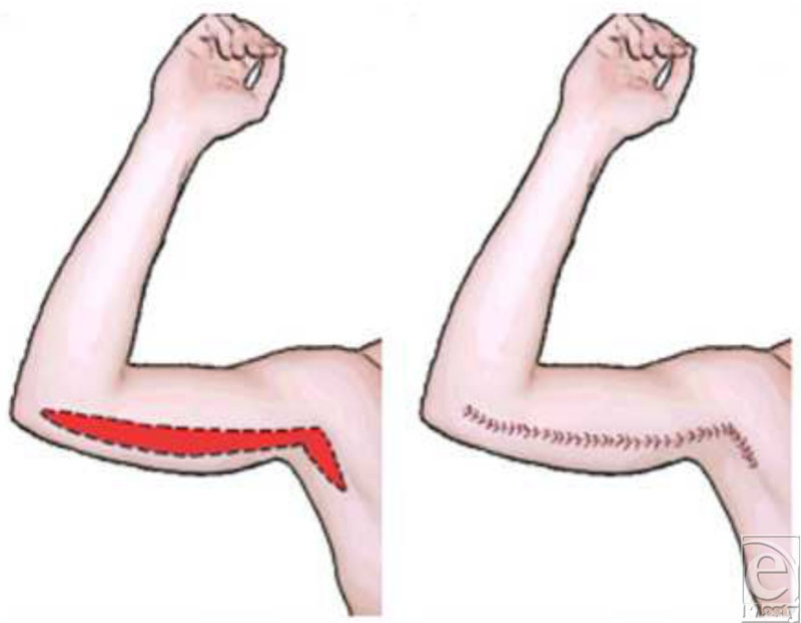
Traditional brachioplasty incision.

Eight fresh cadaver arms were dissected under loupe magnification. After transecting the pectoralis major muscle, the brachial plexus was dissected from proximal to distal to evaluate the branching points of the cutaneous nerves and to avoid injury. Dissections began laterally to preserve cutaneous branches of MBCN. Measurements were taken from the medial epicondyle to the cutaneous branches off of the main nerve. Additional measurements were taken from the medial epicondyle to the acromioclavicular joint. The location of the nerve's course about the basilic vein was also noted (Fig 1).

## RESULTS

There is variability in the course of the MBCN. The MBCN comes off the medial cord of the brachial plexus. All dissections demonstrated the MBCN to course with the basilic vein. At about 7.8 ± 0.6 cm proximal to the medial epicondyle, there is an arborization of 2 to 4 cutaneous branches. This nerve sends branches through the muscular fascia across the ulnar nerve to skin of the medial arm at about 15.3 ± 1.0 cm proximal to the medial epicondyle. In most cadavers, this was found in the midportion of the arm. The average distance from the medial epicondyle to the acromioclavicular joint was 31 ± 3.2 cm (Table 1).

## DISCUSSION

**Figure 2:**
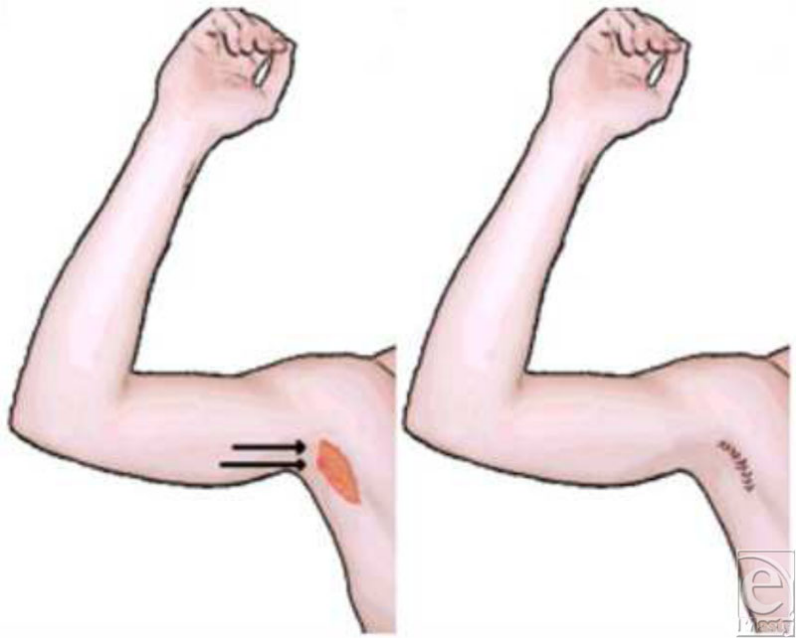
Minibrachioplasty incision and closure.

Early brachioplasty procedures were done with simple, short elliptical excisions that achieved only fair corrections. As the procedure was refined, brachioplasty results improved when emphasis was placed on the correction of the middle to upper third of the arm, where the soft tissue laxity is usually most pronounced (Fig 2). Further improvement occurred after surgeons recognized the importance of secure tightening of the superficial fascial system, as emphasized by Lockwood.^4^ Suturing of the superficial fascial system results in a smoother contour, tighter closure, and a finer scar. With adjunctive liposuction, brachioplasty results improved further because liposuction helps contour and debulk the arm and loosens the subcutaneous tissue plane, making the flap dissection easier.^2^

Some patients undergoing brachioplasty present because of normal aging while others present secondary to weight loss. Determining the appropriate approach depends on the purpose of the reconstruction and the degree of skin laxity. Traditional brachioplasty procedures involve incisions that run from the medial epicondyle to the axilla. This incision is generally placed slightly posterior to the medial bicipital groove to minimize appearance of the scar. Caution is advised when elevating cutaneous tissues as they have close proximity to the ulnar nerve and terminal branches of the MACN and the MBCN. These cutaneous nerves provide pressure and pain sensation to the medial arm and elbow. Injury to this cutaneous system has resulted in the development of pressure ulcers over the elbow in some cases.^7^ Injury to this cutaneous system has resulted in the development of pressure ulcers over the elbow in some cases.^7^

**Figure 3:**
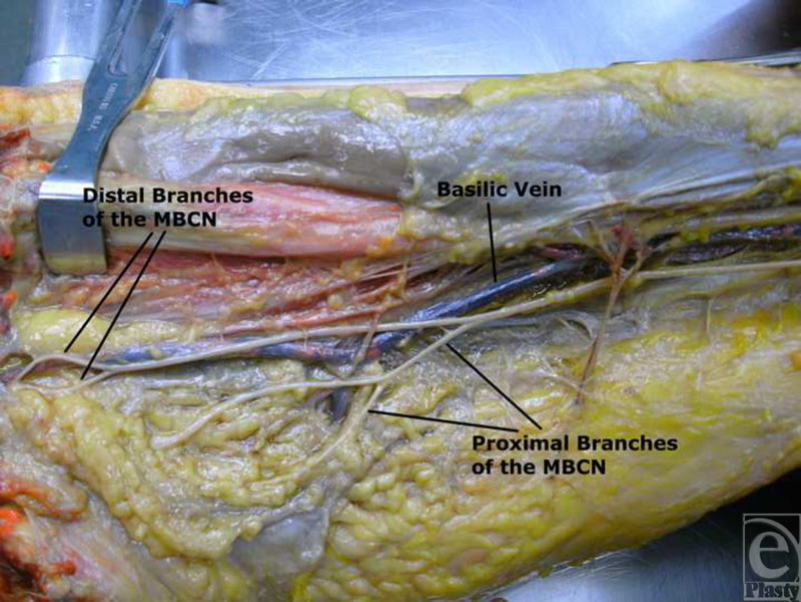
Dissection of the upper arm. MBCN indicates medial brachial cutaneous nerve.

Minibrachioplasty techniques coupled with liposuction offer similar contouring. The entirety of the scar is placed in the axilla to minimize the outward appearance of a scar on visible skin. While the scarring and direction of resection are more transversely oriented, the liposuction cannula can potentially injure these nerves or denervate their corresponding dermatomes, leading to a transient or permanent anesthesia over the medial arm (Fig 3). It is also worth noting that although the aesthetic outcome can be more favorable with the minibrachioplasty in the appropriate patient population, the surgeon is more limited in the contouring of the excess skin than with a traditional brachioplasty.

Regardless of the technique used, care must be taken in elevating flaps at around 7 cm and 15 cm proximal to the medial epicondyle as this corresponds to the approximate location of the arborization points of the MBCN. Preserving these nerves will help prevent the morbidity associated with the anesthetic areas of these dermatomes. Placing the incision slightly more posteriorly can also aid in avoiding these nerves.

While several techniques exist to manage excess skin of the arms and axilla, all potentially endanger the delicate nerves of the region. In their retrospective review of 40 bilateral brachioplasty operations performed over 16 years at the Mayo Clinic, Knoetgen and Moran identified 2 patients with cutaneous nerve injuries in the arm secondary to surgery. These patients can develop painful neuromas. Their study suggests an approximately 5% risk of nerve injury during surgery.^7^ Knowledge of the anatomy of the cutaneous nerves can enhance our ability to perform these contouring procedures and exercise caution as newer techniques evolve.

## CONCLUSIONS

The MBCN runs with the basilic vein and sends 2 to 4 branches to the skin 7 cm proximal to the medial epicondyle. Another 3 to 5 branches pierce the fascia to innervate the skin at about 15 cm proximal to the medial epicondyle. As more patients utilize various weight loss strategies, the plastic surgeon will be challenged to effectively manage the excess skin. Finding solutions that provide satisfactory aesthetic outcomes, while minimizing morbidity and complications, is the aim of the brachioplasty surgery. Placing the incisions more posteriorly can provide similar acceptable aesthetic outcome while minimizing the morbidity associated with nerve injury.

## Figures and Tables

**Table 1:** Locating the medial brachial cutaneous nerve*

Specimen	ME-AC joint, cm	ME–distal branches, cm	ME–proximal branches, cm	No. of distal branches	No. of proximal branches
1	24	6.5	13.0	3	3
2	30	7.4	16.0	2	5
3	29	7.8	15.0	2	3
4	31	8.0	15.5	4	4
5	33	7.8	15.8	2	3
6	35	8.6	16.2	3	5
7	31	8.0	15.2	3	4
8	32	8.2	15.8	4	4
Mean	31	7.8	15.3	2.9	3.9
SD	3.2	0.6	1.0	0.8	0.8

*ME indicates medial epicondyle; AC, acromioclavicular joint.
